# Rheumatic Myocarditis: A Poorly Recognized Etiology of Left Ventricular Dysfunction in Valvular Heart Disease Patients

**DOI:** 10.3389/fcvm.2021.676694

**Published:** 2021-06-10

**Authors:** Vitor Emer Egypto Rosa, Mariana Pezzute Lopes, Guilherme Sobreira Spina, Jose Soares Junior, David Salazar, Cristhian Espinoza Romero, Marcos Pita Lottenberg, Antonio de Santis, Lucas José Neves Tachotti Pires, Luis Fernando Tonello Gonçalves, Joao Ricardo Cordeiro Fernandes, Roney Orismar Sampaio, Flavio Tarasoutchi

**Affiliations:** Heart Institute (InCor) Clinical Hospital, University of São Paulo, São Paulo, Brazil

**Keywords:** myocarditis, rheumatic fever, rheumatic heart disease, valvular heart disease, heart failure

## Abstract

**Background:** Heart failure occurs in ~10% of patients with acute rheumatic fever (RF), and several studies have shown that cardiac decompensation in RF results primarily from valvular disease and is not due to primary myocarditis. However, the literature on this topic is scarce, and a recent case series has shown that recurrent RF can cause ventricular dysfunction even in the absence of valvular heart disease.

**Methods:** The present study evaluated the clinical, laboratory and imaging characteristics of 25 consecutive patients with a clinical diagnosis of myocarditis confirmed by 18F-FDG PET/CT or gallium-67 cardiac scintigraphy and RF reactivation according to the revised Jones Criteria. Patients underwent three sequential echocardiograms at (1) baseline, (2) during myocarditis and (3) post corticosteroid treatment. Patients were divided according to the presence (Group 1) or absence (Group 2) of reduced left ventricular ejection fraction (LVEF) during myocarditis episodes.

**Results:** The median age was 42 (17–51) years, 64% of patients were older than 40 years, and 64% were women. Between Group 1 (*n* = 16) and in Group 2 (*n* = 9), there were no demographic, echocardiographic or laboratory differences except for NYHA III/IV heart failure (Group 1: 100.0% vs. Group 2: 50.0%; *p* = 0.012) and LVEF (30 [25–37] vs. 56 [49–62]%, respectively; *p* < 0.001), as expected. Group 1 patients showed a significant reduction in LVEF during carditis with further improvement after treatment. There was no correlation between LVEF and valvular dysfunction during myocarditis. Among all patients, 19 (76%) underwent 18F-FDG PET/CT, with a positive scan in 68.4%, and 21 (84%) underwent gallium-67 cardiac scintigraphy, with positive uptake in 95.2%, there was no difference between these groups.

**Conclusion:** Myocarditis due to rheumatic fever reactivation can cause left ventricular dysfunction despite valvular disease, and it is reversible after corticosteroid treatment.

## Introduction

Rheumatic fever is a prevalent disease, mainly in low- and middle-income countries but also in specific populations in developed countries. Data on the prevalence of rheumatic fever are probably underreported due to (a) the cost of screening, (b) difficulties in acute rheumatic disease diagnosis and (c) data on surgery or mortality representing rheumatic fever incidence from 2 decades ago. Chronic valvular heart disease is the most feared consequence of rheumatic fever, leading to a decreased quality of life, hospitalizations, and surgical procedures, primarily in young adults (1–5).

Heart failure occurs in ~10% of patients with acute rheumatic fever with carditis and is described mainly during rheumatic fever reactivation (6–9). Valvulitis due to the involvement of the endocardium is the predominant manifestation of rheumatic fever carditis despite myocarditis and pericarditis occurrence. Furthermore, several studies have shown that heart failure in rheumatic fever patients results solely from valvular disease and is not due to primary myocarditis (10–14).

A recent case series of patients with predominantly rheumatic valvular disease undergoing heart transplantation showed that 27.7% had non-diagnosed myocarditis leading to refractory heart failure and ultimately heart transplantation. These patients had normofunctional valve prostheses and left ventricular dysfunction, suggesting that recurrent rheumatic fever may cause subacute myocarditis, a condition difficult to diagnose (15).

The aim of the present study was to evaluate the clinical, laboratory, and echocardiographic profiles of patients with rheumatic fever reactivation and clinical diagnosis of myocarditis, confirmed using fluorine-18-fluorodeoxyglucose positron emission tomography (18F-FDG PET/CT) or gallium-67 cardiac scintigraphy.

## Materials and Methods

### Study Protocol and Population

This was a single-center, retrospective study assessing 25 consecutive patients between 2005 and 2020 with a diagnosis of rheumatic fever reactivation according to the revised Jones Criteria (16). Because the inhabitants of Brazil are considered a high-risk population (4), we selected the following major manifestations in this study: (i) carditis; (ii) monoarthritis, polyarthritis, or polyarthralgia; (iii) chorea; (iv) erythema marginatum; and (v) subcutaneous nodules. The minor manifestations were as follows: (i) monoarthralgia; (ii) fever; (iii) erythrocyte sedimentation rate ≥30 mm/h and/or C-reactive protein (CRP) ≥3.0 mg/dL; and (iv) prolonged PR interval. The presence of Aschoff bodies in the histological examination was considered a definitive criterion for rheumatic fever reactivation.

Rheumatic carditis diagnosis was confirmed in patients with at least one of the four clinical findings: (i) significant murmur (*n* = 4), (ii) cardiac enlargement (*n* = 16), (iii) cardiac decompensation (*n* = 4), or (iv) pericardial friction rub or effusion (*n* = 1) (5). To corroborate myocardial involvement, patients underwent 18F-FDG PET/CT or gallium-67 cardiac scintigraphy. Patients without myocardium involvement in both imaging tests were excluded from the present analysis. Differential diagnoses of myocardial involvement were assessed and excluded. Clinical data included age, sex, symptoms, medications, documented diagnosis of traditional cardiovascular risk factors, and comorbidities, such as hypertension, diabetes mellitus, and coronary artery disease. Patients also underwent laboratory tests and at least three sequential echocardiograms at (1) baseline, (2) during myocarditis, and (3) post corticosteroid treatment. To understand the impact of carditis on left ventricular function, patients were divided into two groups according to the left ventricular ejection fraction course during myocarditis episodes as follows:

- Group 1: reduction of left ventricular ejection fraction during the myocarditis episode;
- Group 2: no reduction in left ventricular ejection fraction during the myocarditis episode.

The study protocol was reviewed and approved by the local institutional ethics committee.

### Echocardiography

All transthoracic Doppler-echocardiographic exams were analyzed in a central echocardiography laboratory at our institution. All exams were performed using a commercially available ultrasound system (Vivid 9, GE Healthcare, Milwaukee, WI, USA or EPIQ 7, Koninklijke Philips N.V., Amsterdam, Noord-Holland, Netherlands). Cardiac chambers were measured using the American Society of Echocardiography standards (17).

Fluorine-18-fluorodeoxyglucose positron emission tomography (18F-FDG PET/CT) imaging: Images were acquired on a positron emission tomography scanner coupled to computed tomography [Gemini TF 64 TOF (Philips Healthcare)] 1 h after intravenous administration of 18F-FDG (370 MBq). To suppress normal myocardial glucose utilization, patient preparation consisted of a high-fat and low- or no-carbohydrate diet 24 h prior to the exam, followed by 8–12 h of fasting. Tomographic reconstruction was performed in both modalities in the axial, sagittal, and coronal planes. FDG uptake in the myocardium was considered positive for inflammation. Visual and quantitative evaluation (standard uptake value, SUV) was performed.

### Gallium-67 Cardiac Scintigraphy

Planar images of the thorax (anterior, lateral, and posterior views) were acquired 72 h after the intravenous injection of 150–185 MBq of gallium-67 citrate using an Infinia gamma camera (GE Healthcare). The intensity of gallium-67 uptake in the heart was compared with that in the ribs and sternum, and any evidence of gallium-67 cardiac uptake (qualitative evaluation) was considered positive for active inflammation.

### Outcomes

The endpoints analyzed were 30-day mortality, vasoactive drug use, surgery during myocarditis and late cardiac death.

### Statistical Analysis

Continuous variables are presented as medians (interquartile ranges), and categorical variables are presented as percentages. The Shapiro-Wilk test was used to test the normality of variables. The Mann-Whitney test was applied for continuous variables, and Fisher's exact test or the Chi-squared test was applied for categorical variables. Generalized estimating equations were used to analyze repeated echocardiographic measures (gamma or binary logistic model as appropriate). The *post-hoc* analysis was performed with a Bonferroni test. Kaplan-Meier curves and log-rank test of the time-to-event data were used to evaluate late cardiac mortality. All tests were two-tailed, and *p* < 0.05 was used to indicate statistical significance. All analyses were conducted using the SPSS statistical package, version 20 (IBM, Armonk, NY).

## Results

### Patient Characteristics

The main baseline clinical and laboratory data are shown in Table 1. Among the 25 patients with myocarditis included in the study, the median age was 42 (17–51) years, 64% of the patients were older than 40 years, and 64% were women. We found a low incidence of comorbidities with the exception of atrial fibrillation (found in 68%), and no patient had coronary artery disease. There were no demographic or laboratory differences between Group 1 (*n* = 16) and Group 2 (*n* = 9). However, Group 1 patients had more NYHA III/IV heart failure than Group 2 patients (100.0 vs. 50.0%, respectively; *p* = 0.012).

**Table 1:** Baseline clinical and laboratory data of the study population.

**Variable**	**Overall (*n* = 25)**	**Group 1 (LVEF reduction during carditis) (*n* = 16)**	**Group 2 (No LVEF reduction during carditis) (*n* = 9)**	***p*-value**
**Clinical data**				
Age, years	42 (17–51)	42 (37–57)	41 (22–51)	0.452
Age ≥40 years	16 (64.0)	11 (68.8)	5 (55.6)	0.821
Female sex	16 (64.0)	11 (68.8)	5 (55.6)	0.821
Hypertension	7 (28.0)	4 (25.0)	3 (33.3)	1.000
Diabetes mellitus	4 (16.0)	3 (18.8)	1 (11.1)	1.000
Dyslipidemia	4 (16.0)	2 (12.5)	2 (22.2)	0.946
Coronary artery disease	–	–	–	–
Previous stroke or TIA	3 (12.0)	2 (12.5)	1 (11.1)	1.000
Atrial fibrillation	17 (68.0)	10 (62.5)	7 (77.8)	0.734
**Symptoms**				
Heart failure NYHA I/II	3 (12.0)	–	3 (37.5)	0.050
Heart failure NYHA III/IV	20 (80.0)	16 (100.0)	4 (50.0)	**0.012**
Arthralgia	3 (12.0)	1 (6.3)	2 (25.0)	0.513
Fever	3 (12.0)	–	3 (33.3)	0.069
**Medications**				
ACE inhibitors or ARB	11 (44.0)	6 (37.5)	5 (55.6)	0.650
β-Blockers	17 (68.0)	12 (75.0)	5 (55.6)	0.580
Antiplatelet agents	5 (20.0)	2 (13.3)	3 (33.3)	0.516
Furosemide	17 (68.0)	12 (75.0)	5 (55.6)	0.580
Spironolactone	10 (40.0)	6 (37.5)	4 (44.4)	1.000
Statins	3 (12.0)	2 (12.5)	1 (11.1)	1.000
Digoxin	9 (36.0)	6 (37.5)	3 (33.3)	1.000
Oral anticoagulation	17 (68.0)	12 (75.0)	5 (55.6)	0.580
Penicillin	9 (36.0)	5 (31.3)	4 (44.4)	0.821
**Laboratory data**				
Hemoglobin, g/dl	12.5 (11.7–13.9)	12.0 (11.5–13.3)	13.5 (12.6–14.0)	0.121
Hematocrit, %	38 (37–41)	37 (34–38)	40 (39–42)	0.083
Leukocytes, /mm3	6,020 (4,110–8,305)	5,690 (388–9,035)	7,540 (4,760–8,372)	0.301
C-reactive protein, mg/dl	6.29 (2.26–23.12)	11.11 (4.24–46.00)	4.36 (1.57–11.20)	0.370
eGFR, ml/min/1.73 m2	77.6 (55.3–84.0)	77.6 (51.4–84.0)	76.4 (59.7–82.6)	0.860

*Values are median (interquartile range), or n (%). ACE indicates angiotensin-converting enzyme; ARB, angiotensin receptor blocker, eGFR, estimated glomerular filtration rate; LVEF, left ventricular ejection fraction, NYHA, New York Heart Association; and TIA, transient ischemic attack. Bold values denote statistical significance*.

### Rheumatic Fever Reactivation Diagnosis

Carditis was considered a major manifestation for all patients. In addition to rheumatic carditis, six patients had two minor manifestations (CRP ≥3.0 mg/dL + fever in three patients; and CRP ≥3.0 mg/dL + monoarthralgia in three patients). Two patients had rheumatic carditis confirmed by myocardial biopsy. Rheumatic carditis was highly suspected in the remaining patients.

### Complementary Tests

**Figure 1:**
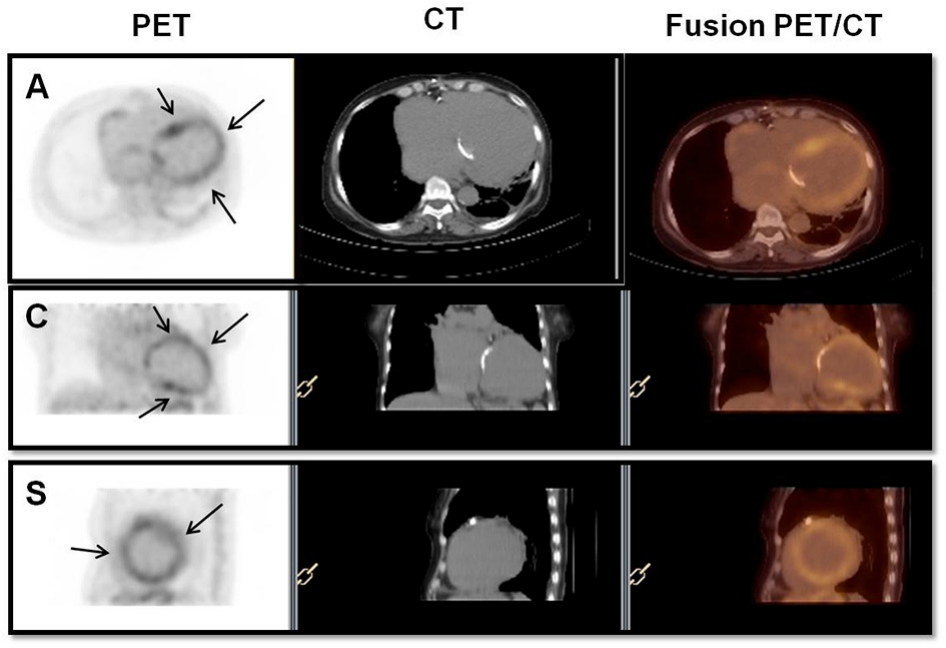
Positive ^18^F-FDG PET/CT scan. Fluorine-18-fluorodeoxyglucose positron emission tomography (18F-FDG PET/CT) (PET, CT, and fusion PET/CT) showing diffuse FDG uptake in the LV myocardium (arrows), indicating active inflammation. The intensity of uptake was discrete, and SUVmax was 3.3. A, axial; C, coronal; S, sagittal; and SUV, standardized uptake value.

**Figure 2:**
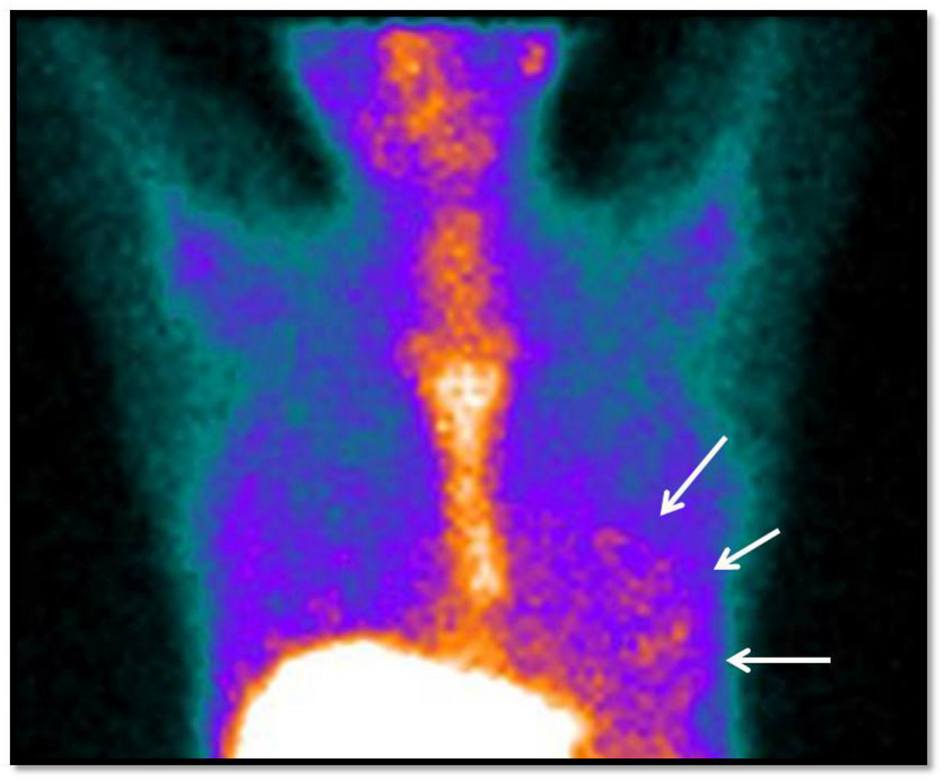
Positive gallium-67 cardiac scintigraphy (anterior view of the thorax). Image obtained 72 h after radiopharmaceutical administration, showing diffuse and mild gallium-67 uptake in cardiac projection (arrows), indicating an inflammatory myocardial process. Physiological gallium-67 uptake was observed in the liver and bones (sternum and ribs).

The main baseline data from echocardiography, 18F-FDG PET/CT and gallium-67 cardiac scintigraphy are shown in Table 2. The criteria for rheumatic carditis were the presence of cardiac enlargement in the 16 patients in Group 1, significant murmur in four patients in Group 2, pericardial effusion in one patient in Group 2 and cardiac decompensation in four patients in Group 2. During myocarditis, Group 1 patients had a lower left ventricular ejection fraction than Group 2 patients (30 [25–37] vs. 56 [49–62]%, respectively; *p* < 0.001), as expected. There was no other significant echocardiographic difference between the groups. Normofunctional valve prostheses, mitral bioprostheses with stenosis or native mitral stenosis were found in 64.0% of patients. In 19 patients, 18F-FDG PET/CT was performed, with positive scans in 68.4% and a median SUV ratio of 4.5 (3.35–8.37), and there was no difference between groups regarding the SUV ratio (Figure 1). Gallium-67 cardiac scintigraphy was performed in 21 patients, with positive uptake in 95.2%, and there was no difference between groups regarding gallium-67 uptake (Figure 2).

**Table 2:** Baseline echocardiographic, 18F-FDG PET/CT and Gallium-67 cardiac scintigraphy data.

**Variable**	**Overall (*n* = 25)**	**Group 1 (LVEF reduction during carditis) (*n* = 16)**	**Group 2 (No LVEF reduction during carditis) (*n* = 9)**	***p*-value**
**Echocardiography during myocarditis**				
LVEF, %	36 (27–53)	30 (25–37)	56 (49–62)	** <0.001**
LVEDD, mm	62 (48–70)	58 (48–68)	65 (47–73)	0.601
LVESD, mm	46 (38–58)	49 (40–58)	45 (32–56)	0.512
LVEDV, mL	145 (93–229)	118 (93–210)	198 (97–268)	0.382
LVESV, mL	76 (55–116)	72 (52–181)	83 (56–115)	1.000
LV mass, g/m^2^	132 (95–165)	123 (96–162)	141 (95–207)	0.773
Pulmonary artery systolic pressure, mmHg	56 (51–64)	57 (52–64)	53 (38–64)	0.301
Moderate/severe rheumatic aortic regurgitation	9 (36.0)	3 (18.8)	6 (66.7)	0.050
Moderate/severe rheumatic mitral regurgitation	14 (58.3)	7 (46.7)	7 (77.8)	0.285
Moderate/severe functional tricuspid regurgitation	17 (73.9)	11 (73.3)	6 (75.0)	1.000
Moderate/severe right ventricular dysfunction	11 (47.8)	9 (60.0)	2 (25.0)	0.245
Normofunctional valve prosthesis, mitral bioprosthesis stenosis or native mitral stenosis	16 (64.0)	11 (68.8)	5 (55.6)	0.821
Pericardial effusion				0.267
Discrete	5 (20.0)	4 (26.7)	1 (12.5)	
Moderate	1 (4.0)	–	1 (12.5)	
**18F-FDG PET/CT**	*N* = 19	*N* = 13	*N* = 6	
Positive scan	13 (68.4)	10 (76.9)	3 (50.0)	0.520
SUV max	4.5 (3.35–8.37)	4.15 (3.12–7.22)	7.64 (3.50–7.64)	0.371
**Gallium-67 cardiac scintigraphy**	*N* = 21	*N* = 13	*N* = 8	
Positive Gallium-67 imaging	20 (95.2)	12 (92.3)	8 (100.0)	1.000
Gallium uptake				0.070
Discrete	12 (60.0)	5 (41.7)	7 (87.5)	
Discrete/moderate	8 (40.0)	7 (58.3)	1 (12.5)	

*Values are median (interquartile range), or n (%).18F-FDG PET/CT indicates fluorine-18-fluorodeoxyglucose positron emission tomography; LV, left ventricular; LVEDD, left ventricular end-diastolic diameter; LVEDV, left ventricular end-diastolic volume; LVEF, left ventricular ejection fraction; LVESD, left ventricular end-systolic diameter; LVESV, left ventricular end-systolic volume; and SUV, standardized uptake value. Bold values denote statistical significance*.

### Sequential Echocardiographic Findings

**Figure 3:**
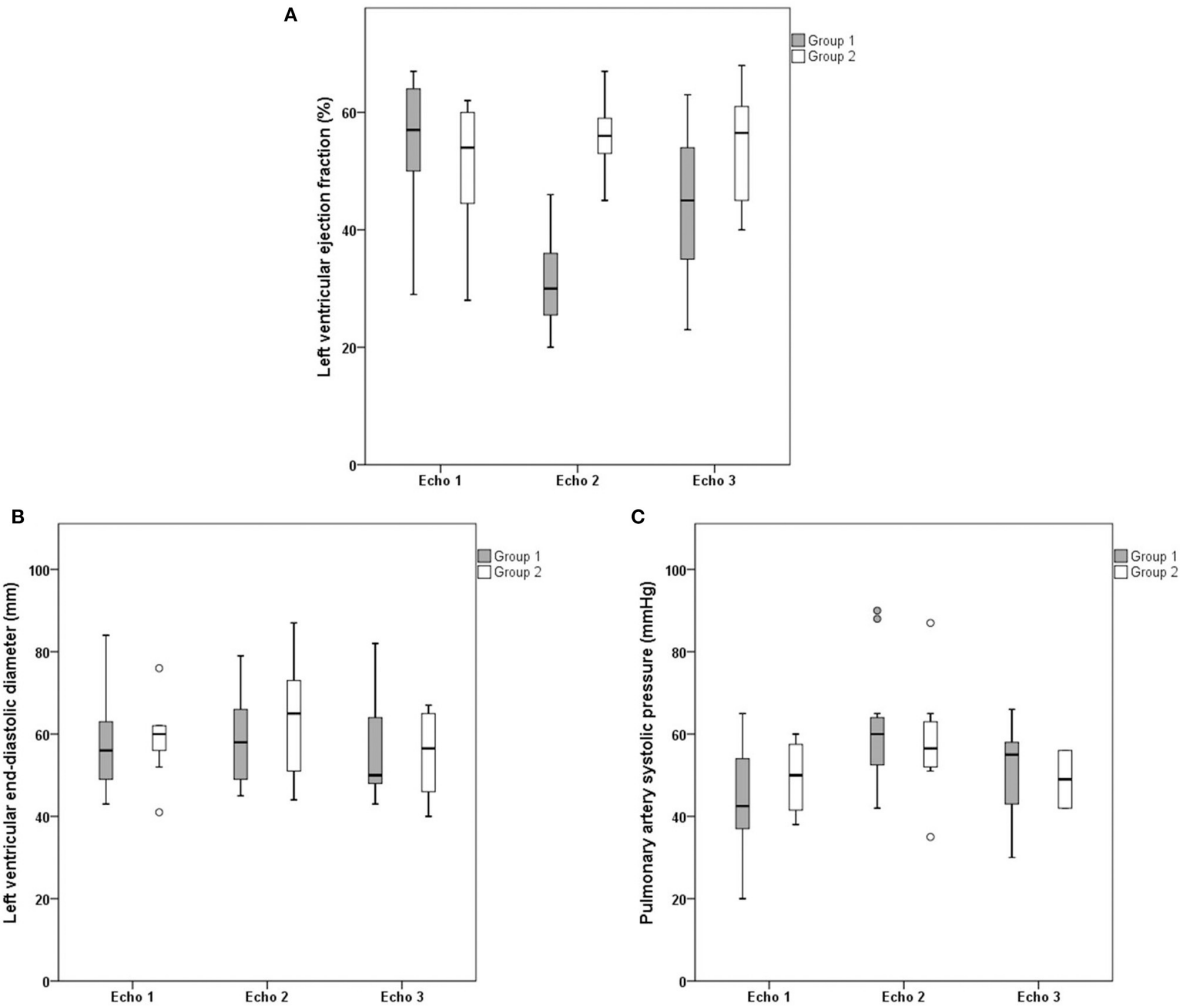
Sequential echocardiographic findings: left ventricular ejection fraction, left ventricular end-diastolic diameter and pulmonary artery systolic pressure. Comparison of baseline echocardiogram (Echo 1), echocardiogram during myocarditis (Echo 2) and postcorticosteroid treatment echocardiogram (Echo 3) between Group 1 (left ventricular ejection fraction reduction during carditis) and Group 2 (no left ventricular ejection fraction reduction during carditis). Comparison of **(A)** left ventricular ejection fraction, **(B)** left ventricular end-diastolic diameter, and **(C)** pulmonary artery systolic pressure. Solid horizontal line indicates mean value. Gray and white boxes indicate 1 SD, and vertical lines indicate highest and lowest mean values.

**Figure 4:**
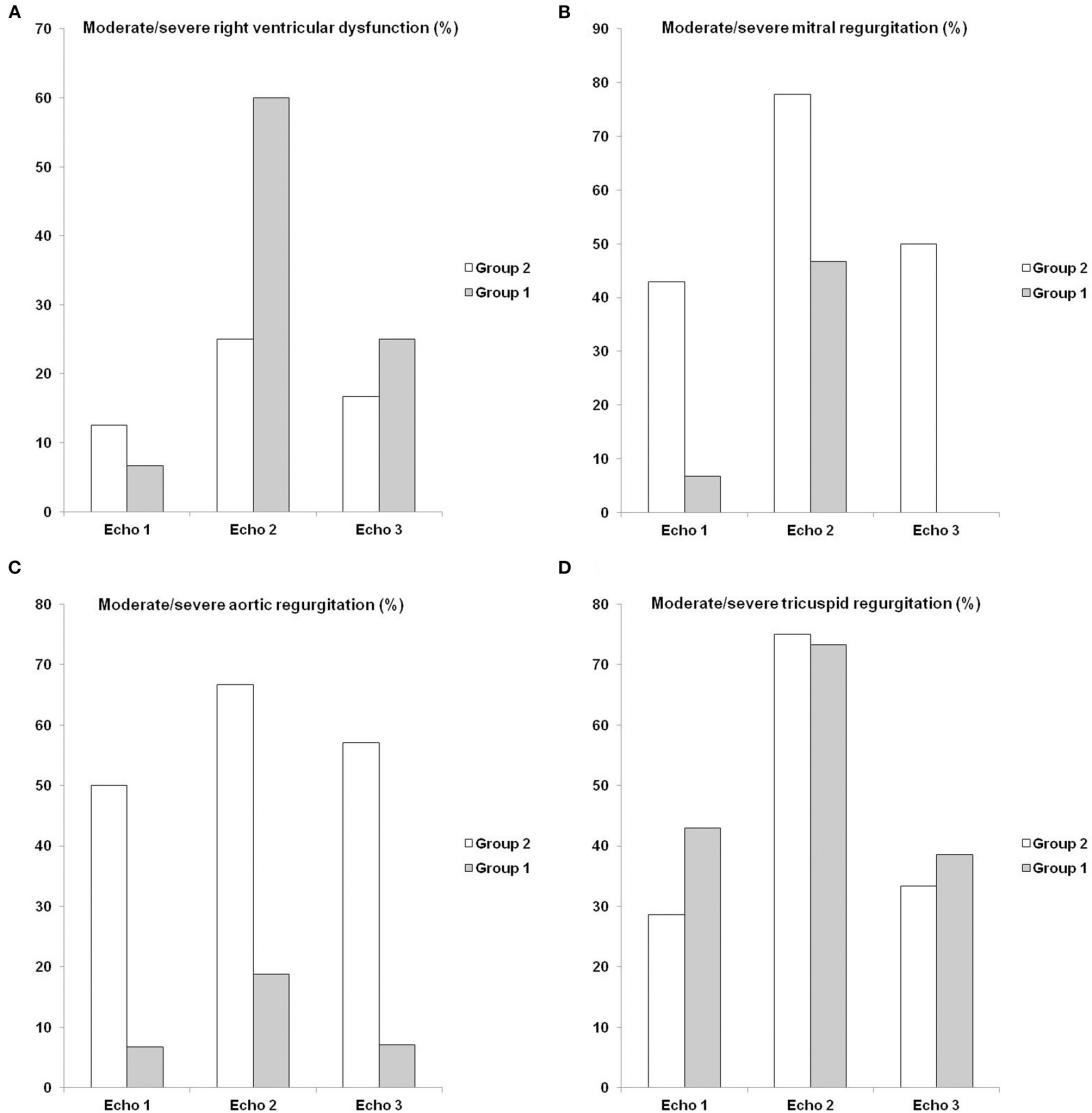
Sequential echocardiographic findings: moderate/severe right ventricular dysfunction, moderate/severe mitral regurgitation, moderate/severe aortic regurgitation and moderate/severe tricuspid regurgitation. Comparison of baseline echocardiogram (Echo 1), echocardiogram during myocarditis (Echo 2) and postcorticosteroid treatment echocardiogram (Echo 3) between Group 1 (left ventricular ejection fraction reduction during carditis) and Group 2 (no left ventricular ejection fraction reduction during carditis). Comparison of **(A)** moderate/severe right ventricular dysfunction, **(B)** moderate/severe mitral regurgitation, **(C)** moderate/severe aortic regurgitation, and **(D)** moderate/severe tricuspid regurgitation. Gray and white bars indicate percentage (%) of each group.

A comparison of Group 1 and Group 2 baseline echocardiography and echocardiography during myocarditis and post-treatment echocardiography is shown in Table 3, Figures 3, 4. There was a difference within subjects in relation to the three echocardiograms and between groups regarding the left ventricular ejection fraction (*p* < 0.001 and *p* = 0.011, respectively). The *post-hoc* analysis showed that differences existed between baseline echocardiography and echocardiography during myocarditis (*p* < 0.001) as well as between echocardiography during myocarditis and post-treatment echocardiography (*p* = 0.020) within Group 1 subjects. When comparing Group 1 to Group 2, differences were related to echocardiography during myocarditis (*p* < 0.001) as previously described. Thus, Group 1 patients showed a significant reduction in left ventricular function during carditis with further improvement after treatment.

**Table 3:** Comparison of baseline echocardiography, echocardiography during myocarditis and post-treatment echocardiography of patients with and without reduction of left ventricular ejection fraction during carditis episode.

	**Group 1 (LVEF reduction during carditis) (*****n*** **=** **16)**			**Group 2 (No LVEF reduction during carditis) (*****n*** **=** **9)**				
**Variable**	**Baseline echocardiography**	**Echocardiography during myocarditis**	**Post-treatment echocardiography**	**Baseline echocardiography**	**Echocardiography during myocarditis**	**Post-treatment echocardiography**	**P (WS)**	**P (BG)**
LVEF, %	57 (49–64)	30 (25–37)	45 (30–54)	54 (39–61)	56 (49–62)	56 (43–62)	** <0.001**	**0.011**
LVEDD, mm	56 (48–64)	58 (48–68)	50 (48–65)	60 (52–62)	65 (47–73)	56 (44–65)	**0.039**	0.754
LVESD, mm	39 (31–48)	49 (40–58)	36 (32–56)	41 (28–50)	45 (32–56)	40 (29–54)	0.053	0.563
LVEDV, mL	154 (97–194)	118 (93–210)	118 (87–224)	187 (16–222)	198 (97–268)	107 (76–191)	0.268	0.822
LVESV, mL	66 (39–90)	72 (52–181)	45 (41–173)	78 (45–127)	83 (56–115)	42 (26–88)	0.240	0.369
LV mass, g/m^2^	102 (93–134)	123 (96–162)	98 (88–135)	132 (86–172)	141 (95–207)	109 (75–169)	0.149	0.632
Pulmonary artery systolic pressure, mmHg	46 (33–58)	57 (52– 64)	55 (42–59)	50 (39–58)	53 (38–64)	49 (42–49)	**0.019**	0.885
Moderate/severe rheumatic aortic regurgitation	1 (6.7)	3 (18.8)	1 (7.1)	4 (50.0)	6 (66.7)	4 (57.1)	0.835	0.053
Moderate/severe rheumatic mitral regurgitation	1 (6.7)	7 (46.7)	–	3 (42.9)	7 (77.8)	3 (50.0)	** <0.001**	** <0.001**
Moderate/severe functional tricuspid regurgitation	6 (42.9)	11 (73.3)	5 (38.5)	2 (28.6)	6 (75.0)	2 (33.3)	**0.005**	0.718
Moderate/severe right ventricular dysfunction	1 (6.7)	9 (60.0)	3 (25.0)	1 (12.5)	2 (25.0)	1 (16.7)	**0.013**	0.663

*Values are median (interquartile range), or n (%). BG indicates between groups; LV, left ventricular; LVEDD, left ventricular end-diastolic diameter; LVEDV, left ventricular end-diastolic volume; LVEF, left ventricular ejection fraction; LVESD, left ventricular end-systolic diameter; LVESV, left ventricular end-systolic volume; and WS, within subjects. Bold values denote statistical significance*.

### Correlation Between Echocardiographic Data During Myocarditis

There was no correlation between left ventricular ejection fraction and moderate/severe rheumatic mitral regurgitation (*p* = 0.375), moderate/severe rheumatic aortic regurgitation (*p* = 0.437), moderate/severe functional tricuspid regurgitation (*p* = 0.320), and moderate/severe right ventricular dysfunction (*p* = 0.053).

### Clinical Outcomes

The clinical outcomes are shown in Table 4. All patients were treated with corticosteroids, and 76.0% were treated during hospitalization. Valvular heart surgery during myocarditis was performed in 12% of patients, and there was no 30-day mortality. The median follow-up was 10.8 (2.6–30.8) months, and late cardiovascular deaths occurred in 20% of patients, with no difference between the groups (log-rank *p* = 0.829). Causes of late death were septic shock (*n* = 2), endocarditis (*n* = 1), cardiogenic shock (*n* = 1), and complications of myocardium biopsy (*n* = 1).

**Table 4:** Clinical outcomes.

**Variable**	**Overall (*n* = 25)**	**Group 1 (LVEF reduction during carditis) (*n* = 16)**	**Group 2 (No LVEF reduction during carditis) (*n* = 9)**	***p*-value**
In-hospital care	19 (76.0)	12 (75.0)	7 (77.8)	1.000
Vasoactive drugs	12 (48.0)	8 (50.0)	4 (44.4)	1.000
Surgery during carditis	3 (12.0)	3 (18.8)	–	0.457
30-day mortality	–	–	–	–
Late cardiovascular death	5 (20.0)	2 (12.5)	3 (33.3)	0.466

*Values are n (%). LVEF indicates left ventricular ejection fraction*.

## Discussion

The main findings of the present study were as follows: (1) rheumatic fever reactivation can cause myocarditis and left ventricular dysfunction in the absence of severe valvular heart disease; and (2) in these cases, the left ventricular ejection fraction improves after corticosteroid treatment.

Rheumatic heart disease is the most common consequence of a prevalent and difficult-to-diagnose disease. Approximately 20% of rheumatic fever patients may present reactivation episodes in 10 years, causing death, hospitalization and worsening of valvular heart disease severity (18). In addition, the diagnosis of acute rheumatic fever is complex. There is no definitive diagnostic test, and clinical criteria show high sensitivity and low specificity (16). These factors, together with the difficulty of accessing health services in low-income countries, explain the relatively low acute-phase diagnostic rate and low number of patients included in studies (1–5).

Regarding rheumatic carditis, diagnostic criteria are vague and depend only on the presence of valvulitis (16). In addition, several studies and guidelines claim that valvular disease is the cause of cardiac decompensation in acute rheumatic fever and not myocardial dysfunction itself (5, 10–14), which raises several questions. It is unknown if patients with valve prostheses have acute rheumatic myocarditis, and if they do, it is unknown if these patients are protected from cardiac decompensation.

An unexpected finding from a previous study refutes this hypothesis. In this previous case series of patients with valvular heart disease undergoing heart transplantation, Aschoff bodies were found in the histological examination of 27.7% of the recipients' hearts. Those patients did not have a prior diagnosis of reactivated rheumatic myocarditis at the time of transplantation, and they had normofunctional valve prostheses and left ventricular dysfunction, suggesting a subacute myocarditis diagnosis (15). Unlike the cited study, all patients in the present study were treated with corticosteroids, including patients with a reduction in the left ventricular ejection fraction. Despite the lack of pathological confirmation of rheumatic fever reactivation, rheumatic myocarditis was an exclusion diagnosis, and the improvement of left ventricular ejection fraction after treatment was highly suggestive of rheumatic fever. Notably, no patient had a history of coronary artery disease or other cardiomyopathy.

All patients underwent confirmation of myocarditis using 18F-FDG PET/CT or gallium-67 cardiac scintigraphy. 18F-FDG PET/CT is a new tool for the diagnosis of inflammation. F-18 2-fluoro2-deoxy-D glucose (18F-FDG) is an analog of glucose, and like glucose, it is taken up by activated inflammatory cells that accumulate at the sites of infection or inflammation. Thus, diffuse myocardial uptake is highly suggestive of myocarditis. Because the heart uses a mixture of free fatty acids and glucose for energy production under normal resting conditions and to obtain information regarding the inflammatory process in the myocardium, it is necessary to inhibit physiological myocardial glucose uptake. For this purpose, we used a preparation that consisted of a high-fat and low- or no-carbohydrate diet 24 h before 18F-FDG administration. Data on the role of 18F-FDG PET/CT in the context of acute rheumatic fever are scarce. However, previous studies have reported low sensitivity but high positive predictive value of 18F-FDG PET/CT in the context of acute rheumatic fever (19, 20). Gallium-67 cardiac scintigraphy is also a marker of cardiac inflammation, and the accuracy of this technique varies according to the etiology of myocarditis (21). Data on the role of gallium-67 cardiac scintigraphy in acute rheumatic fever patients have shown good sensitivity and positive predictive value for the diagnosis and evaluation of the therapeutic results (22). Most of the 20 patients undergoing gallium-67 cardiac scintigraphy showed discrete uptake, which confirmed previous studies demonstrating that rheumatic fever is characterized predominantly by interstitial inflammatory changes with minimal damage to myocardial cells and, thus, with low levels of troponin T or I (12, 23, 24). It is important to note that 18F-FDG PET/CT or gallium-67 cardiac scintigraphy was not mandatory for rheumatic carditis diagnosis. These procedures were used only to demonstrate that active inflammation occurs not only in the endocardium but also in the myocardium. Rheumatic carditis is an exclusion criterion, and the improvement of symptoms and reverse cardiac remodeling after corticosteroid treatment retrospectively reinforced the diagnosis.

Our patients differed from those with acute rheumatic myocarditis reported in the literature. Most previous studies have included first episodes in children, while the patients in our study had reactivation and were mostly (64%) older than 40 years (10–14). In addition, moderate/severe mitral regurgitation showed an increased incidence during myocarditis episodes. However, unlike the literature, there was no correlation between mitral regurgitation and left ventricular ejection fraction in the present study, and only three patients required surgical treatment during carditis. Another important aspect was the high prevalence of atrial fibrillation (68%). Despite the low median age of the studied population (42 [17–51] years), chronic rheumatic heart disease is associated with an increased prevalence of atrial fibrillation, ranging from 4.3 to 79.9% (25).

The present study had several limitations. First, this was a single-center study with a relatively small number of patients (albeit large for this clinical entity). Second, this was a retrospective study with all inherent bias due to its nature. Although left ventricular dysfunction was not related to valvular disease itself in most of patients, it is difficult to rule-out valvular disease as a cause of heart failure in the these patients due to the high prevalence of atrial fibrillation and due to the study design. Unfortunately, we were also unable to evaluate some important data, e.g., electrocardiogram analysis. Third, patients who did not undergo 18F-FDG PET/CT or gallium-67 cardiac scintigraphy and those whose tests were negative were excluded from the analysis. This bias may have contributed to underestimating 30-day mortality.

In conclusion, this study showed that myocarditis due to rheumatic fever reactivation may cause left ventricular dysfunction, which is reversible after corticosteroid treatment. In addition, heart failure was not related to valvular disease itself in 64% of patients. These findings contradict the statement that heart failure in the acute-phase of carditis only occurs in patients with severe valvular lesions. However, due to the inherent limitations of the present study, further prospective research is needed to confirm these findings.

## Data Availability Statement

The datasets presented in this article are not readily available because the data, analytic methods, and study materials will not be made available to other researchers for purposes of reproducing the results or replicating the procedure. Requests to access the datasets should be directed to Vitor Rosa, vitoremer@yahoo.com.br.

## Ethics Statement

The studies involving human participants were reviewed and approved by CAPPesq - Comissão de Ética para Análise de Projetos de Pesquisa do HCFMUSP. Written informed consent for participation was not required for this study in accordance with the national legislation and the institutional requirements.

## Author Contributions

VR, GS, JS, LG, AdS, JF, and RS: design of the study. MLop, DS, CR, and MLot: data collection. VR and RS: data analysis. VR: drafting. VR, MLop, GS, JS, AdS, LP, JF, RS, and FT: approval of the final version. All authors contributed to the article and approved the submitted version.

## Conflict of Interest

The authors declare that the research was conducted in the absence of any commercial or financial relationships that could be construed as a potential conflict of interest.
